# Excess Branched-Chain Amino Acids Suppress Mitochondrial Function and Biogenic Signaling but Not Mitochondrial Dynamics in a Myotube Model of Skeletal Muscle Insulin Resistance

**DOI:** 10.3390/metabo14070389

**Published:** 2024-07-17

**Authors:** Lindsey R. VanDerStad, Emily C. Wyatt, Roger A. Vaughan

**Affiliations:** Department of Health and Human Performance, High Point University, High Point, NC 27268, USA; lvanders@highpoint.edu (L.R.V.); ewyatt@highpoint.edu (E.C.W.)

**Keywords:** leucine, isoleucine, valine, mitochondrial function, skeletal muscle, insulin resistance

## Abstract

Branched-chain amino acids (BCAA) are correlated with severity of insulin resistance, which may partially result from mitochondrial dysfunction. Mitochondrial dysfunction is also common during insulin resistance and is regulated in part by altered mitochondrial fusion and fission (mitochondrial dynamics). To assess the effect of BCAA on mitochondrial dynamics during insulin resistance, the present study examined the effect of BCAA on mitochondrial function and indicators of mitochondrial dynamics in a myotube model of insulin resistance. C2C12 myotubes were treated with stock DMEM or DMEM with additional BCAA at 0.2 mM, 2 mM, or 20 mM to achieve a continuum of concentrations ranging from physiologically attainable to supraphysiological (BCAA overload) both with and without hyperinsulinemia-mediated insulin resistance. qRT-PCR and Western blot were used to measure gene and protein expression of targets associated with mitochondrial dynamics. Mitochondrial function was assessed by oxygen consumption, and mitochondrial content was measured using mitochondrial-specific staining. Insulin resistance reduced mitochondrial function, peroxisome proliferator-activated receptor gamma coactivator 1-alpha mRNA, and citrate synthase expression mRNA, but not protein expression. Excess BCAA at 20 mM also independently reduced mitochondrial function in insulin-sensitive cells. BCAA did not alter indicators of mitochondrial dynamics at the mRNA or protein level, while insulin resistance reduced mitochondrial fission protein 1 mRNA, but not protein expression. Collectively, BCAA at excessively high levels or coupled with insulin resistances reduce mitochondrial function and content but do not appear to alter mitochondrial dynamics under the tested conditions.

## 1. Introduction

A consistent link between elevated branched-chain amino acids (BCAA) and severity of insulin resistance has been observed [1,2,3,4]. Evidence also exists that BCAA improve mitochondrial function [5,6,7,8], yet mitochondrial dysfunction is a proposed mechanism by which BCAA may promote insulin resistance. Previously, we assessed the effects of varied levels of BCAA both with and without concurrent insulin resistance and found BCAA at physiologically attainable levels did not inhibit mitochondrial function, and in fact, increased mitochondrial content [9]. Conversely, during insulin resistance, supraphysiological BCAA reduced mitochondrial function and related gene expression [9]. However, a limitation of our paper was the lack of exploration into the effect of BCAA with and without insulin resistance on mitochondrial dynamics. Insulin resistance has previously been linked to altered mitochondrial dynamics [10,11] and represents another therapeutic opportunity to improve insulin resistance [12].

Conversely, less is known about the effects of BCAA on mitochondrial dynamics, though some evidence suggests amino acids including leucine may alter mitochondrial dynamics [13,14]. In cultured myotubes, leucine had varying effects on Optic atrophy 1 (OPA1) protein expression in the presence and absence of either rapamycin and/or palmitate [14]. ZSF1 rats given normal chow enriched with 3% leucine displayed increased FIS1 and MFN2 protein expression in soleus muscle [15]. Another study investigated the effects of BCAA on mitochondrial dynamics in muscle in the context of detraining and found that BCAA treatment rescued COXIV and mitochondrial enzyme activities to control (trained only), which was otherwise depressed in the detrained only group [16]. And while Matsunaga et al. did not see any restoring effect of BCAA on detraining suppression of PGC-1α or SIRT1 expression, they observed reduced DRP1 expression in detrained mice supplemented with BCAA, but not mice that only received detraining [16].

Collectively, mitochondrial function is implicated in the pathology of insulin resistance [17,18], and alterations in mitochondrial dynamics may represent some aspects of mitochondrial dysfunction during insulin resistance [10,11,12]. Given we previously showed that BCAA overload reduced mitochondrial function in a way dependent on insulin resistance [9], we sought to build on our previous work on the interaction between insulin resistance, BCAA, and mitochondrial dysfunction by examining the effect of varied levels of BCAA both with and without concurrent insulin resistance on mitochondrial dynamics in a myotube model of insulin resistance. We hypothesized that increasing BCAA treatment would result in increased fission-related signaling with decreased fusion-related signaling. We also hypothesized that insulin resistance would be associated with a shift toward mitochondrial fission [11], and that the addition of BCAA treatment would further increase fission-related signaling in the presence of insulin resistance. Such findings would provide additional insight into the relationship between elevated circulating amino acids, insulin resistance, and mitochondrial dysfunction.

## 2. Materials and Methods

### 2.1. Cell Culture

C2C12 mouse myoblasts from ATCC (Manassas, VA, USA) were cultured in Dulbecco’s Modified Eagle’s Medium (DMEM) containing 4500 mg/L glucose and supplemented with 20% heat-inactivated fetal bovine serum and 100 U/mL penicillin and 100 µg/mL streptomycin in a humidified 5% CO_2_ atmosphere at 37 °C. Cells were seeded and grown to confluency with the growth media changed every 2 to 3 days. Differentiation was accomplished by replacing growth media with DMEM supplemented with 2% horse serum 100 U/mL penicillin and 100 µg/mL streptomycin for 4–6 days. A BCAA mixture (Optimum Nutrition, Downers Grove, IL, USA) was dissolved in pre-warmed differentiation media at 20 mM (leucine content) followed by filter sterilization. Cells were then treated with the BCAA mixture in differentiation media with either physiological concentrations, 0.2 mM or 2 mM, or supraphysiological concentrations, represented by 20 mM, for 6 days [9]. This concentration range was chosen to capture the continuum of physiological and supraphysiological levels of BCAA. Insulin resistance was accomplished by adding insulin at 100 nM during the 6-day treatment period (for insulin-resistant groups). To assess the effect of each condition on cell viability following 6-day treatment, media containing each treatment condition was replaced with stock media containing 5% WST-1 substrate, and absorbance at 450 nm was measured every 5 min for 90 min.

### 2.2. Quantitative Real Time Polymerase Chain Reaction (qRT-PCR)

Following treatment, total mRNA was extracted using the Trizol method and quantified (via NanoDrop from Thermo Fisher, Wilmington, DE, USA), and cDNA was synthesized using the iScript cDNA Synthesis Kit from Bio-Rad (Hercules, CA, USA) according to the manufacturer’s instructions. PCR primers were synthesized by Integrated DNA Technologies (Coralville, IA, USA). Amplification of target genes was normalized to the housekeeping gene TATA box binding protein (Tbp) as previously performed [9]. qRT-PCR reactions were performed using the CFX Connect System from Bio-Rad (Hercules, CA, USA). SYBR Green-based PCR was performed using final primer concentrations at 3.75 µM in a total volume of 10 µL per well. The following cycling parameters were used: 95 °C for 3 min followed by 40 cycles of 95 °C for 15 s, and 60 °C for 30 s. qRT-PCR reactions were performed using 3 individual replicates per treatment condition and repeated across 2 independent experiments (*n* = 6 per group). Relative quantification was determined via ^ΔΔ^Ct method. Primer sequences are presented in Table S1.

### 2.3. Immunoblotting

Following treatment, cells were either collected (citrate synthase (CS) and Optic atrophy 1 (OPA1)) or subjected to 30-min stimulation with insulin at 100 nM (pAkt, Akt, Mitofusin 1 (MFN1), Mitofusin 2 (MFN2), Dynamin-related protein 1 (DRP1), and mitochondrial fission protein 1 (FIS1)). Whole cell lysates were prepared by harvesting the cells on ice in RIPA buffer supplemented with protease inhibitor mix (0.1%), followed by incubation on ice for 60 min. Insoluble material was removed, and protein concentrations were determined by Bradford assay. Total protein (50 μg per sample) was size-separated by 10% sodium dodecyl sulfate polyacrylamide gel electrophoresis (SDS-PAGE) and electro-transferred to PVDF membranes. After blocking in TBST-5% non-fat milk powder for 1 h, membranes were probed at 4 °C overnight with primary antibodies (see Table S2 for antibody details and dilutions) in TBST-5% non-fat milk powder. Relative signal intensities were normalized to total Akt or β-Actin and quantified using Image Lab from Bio-Rad (Hercules, CA, USA). Bound antibodies were detected by horseradish peroxidase-conjugated secondary antibodies from AbCam (Cambridge, MA, USA) at a dilution of 1:5000 in TBST-5% non-fat milk powder for 1 h at room temperature while shaking. Protein signal intensities were determined by chemiluminescence using the Clarity Western ECL substrate kit from Bio-Rad (Hercules, CA, USA) and imaged using the ChemiDoc Touch from Bio-Rad (Hercules, CA, USA). Blots were performed using 4 replicates per condition.

### 2.4. Seahorse Metabolic Assays

Cells were seeded into Seahorse XFe96 culture plates, differentiated, and treated as described above. Media were then replaced with buffer-free XF Assay Media obtained from Agilent Technologies (Santa Clara, CA, USA) containing glucose at 25 mM, pyruvate at 1 mM, and glutamine at 2 mM. Following incubation, baseline measurements of oxygen consumption rate (OCR) and extracellular acidification rate (ECAR) were recorded as indicators of basal oxidative metabolism and glycolytic metabolism, respectively. Following basal measurements, each well was infused with oligomycin (an inhibitor of ATP synthase) at a final concentration of 2 μM to induce maximal glycolytic metabolism. Cells were then exposed to carbonyl cyanide p-[trifluoromethoxy]-phenyl-hydrazone (FCCP) at 2 μM to uncouple electron transport and induce peak OCR. Maximal respiration measurements were followed by the injection of rotenone at 1 μM to reveal non-mitochondrial respiration. The Seahorse XFe96 Analyzer was run using a 6-min cyclic protocol command (mix for 3 min and measure for 3 min). MitoStress assays included *n* = 11–12 per group repeated with 2 independent experiments for *n* = 22–24 per group for the final analysis. States of mitochondrial metabolism were calculated by subtracting non-mitochondrial respiration from basal or FCCP-induced peak mitochondrial oxygen consumption. Wells with negative OCR values or no response to injection were removed from the final analysis.

### 2.5. Fluorescent Staining and Microscopy

Immediately following the Seahorse metabolic assay described above, cells were fixed using 3.7% formaldehyde at 37 °C with a 5% CO_2_ atmosphere. The fixing agent was then removed, and cells were stained with DAPI at 0.5 µM in PBS and fluorescence was measured at 360/460 nM (Figure S1). Given that significant differences were observed for nuclei staining, DAPI normalized data are presented for metabolic measurements. To reveal mitochondrial content, cells were then stained with 100 µM nonyl acridine orange (NAO) (Fremont, CA, USA) in PBS and incubated in the dark at room temperature for 10 min. Fluorescence was then measured using 485/525 nm excitation/emission. Neutral lipid content was measured using Nile Red staining at in 10 µM PBS with 1% DMSO vol/vol using 530/645 nm excitation/emission. All fluorescent measurements were made in triplicate and the average (less background) analyzed with *n* = 11–12 per group repeated with two independent experiments with *n* = 22–24 per group for the final analyses. Following fluorescent quantification, cells were imaged using the 20× objective using the Motic AE31E inverted microscope and Moticam Pro 252B (Causeway Bay, Hong Kong).

### 2.6. Statistical Analyses

Data are presented as dot plot and were analyzed using two-way ANOVA with subsequent group comparisons with Bonferroni’s correction. Mitostress assay time trials were analyzed using a three-way repeated measures ANOVA (with time as the lone repeated measure factor) with subsequent group comparisons with Bonferroni’s correction. Values of *p* < 0.05 were used to identify significant differences between groups.

## 3. Results

### 3.1. Effect of BCAA Treatment on Insulin Sensitivity

**Figure 1:**
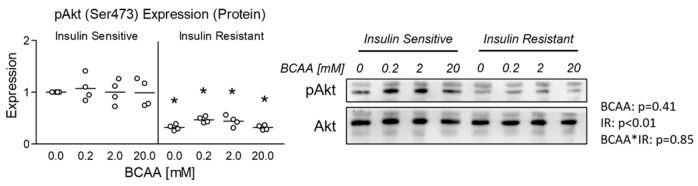
Effect of 6-day BCAA with and without insulin resistance on insulin sensitivity. Effect of BCAA 2:1:1 (leucine/isoleucine/valine) at varied concentrations (0, 0.2, 2, or 20 mM normalized to leucine content) for 6 days with and without insulin resistance (IR) on myotube insulin sensitivity indicated by pAkt(Ser473):Akt expression following stimulation with 100 nM insulin for 30 min. Notes: Data were analyzed using two-way ANOVA with Bonferroni’s correction for multiple comparisons between individual groups. * Indicates *p* < 0.05 between insulin-sensitive and insulin-resistant cells at the specified BCAA concentration. Protein expression was normalized to total Akt using 4 replicates per group with *n* = 4 for the final analysis.

We began our experiments by verifying insulin resistance as evidenced by reduced pAkt expression in all insulin-resistant groups following insulin stimulation (Figure 1). Interestingly, no effect of BCAA was observed at any concentration in either insulin-sensitive or insulin-resistant cells (Figure 1).

### 3.2. Effect of BCAA Treatment on Mitochondrial Function and Content

**Figure 2:**
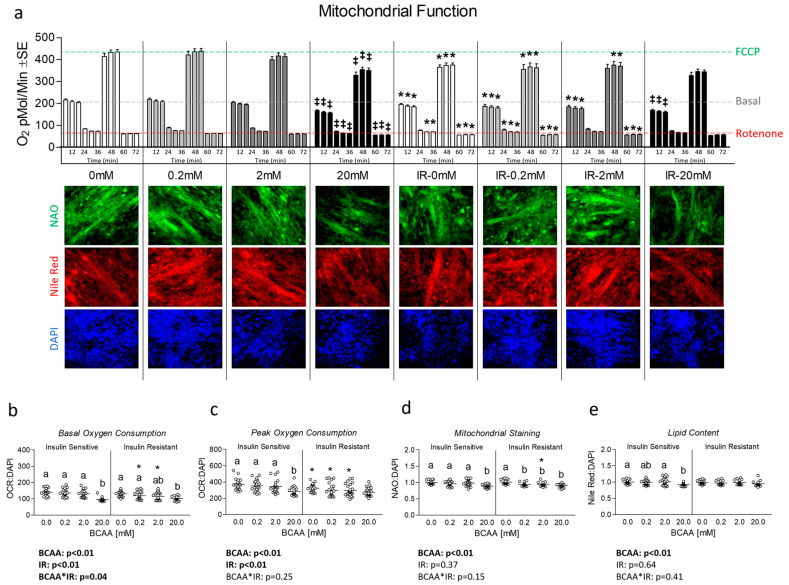
Effect of 6-day BCAA treatment with and without insulin resistance on mitochondrial function and content. (**a**) Time trial of mitochondrial function (Seahorse Mitostress assay) following treatment of myotubes with BCAA 2:1:1 (leucine/isoleucine/valine) at varied concentrations (0, 0.2, 2, or 20 mM normalized to leucine content) for 6 days with and without insulin resistance (IR). (**b**,**c**) Basal and peak mitochondrial function following removal of non-mitochondrial respiration and normalization to nuclei content (Figure S1), respectively. (**d**) Mitochondrial staining following treatment as described in “a”. (**e**) Lipid staining following treatment as described in “a”. Notes: For the time trial in “a”, a three-way repeated measures ANOVA with time as the lone repeated measures factor was used to analyze the data with Bonferroni’s correction to correct for multiple comparisons. ‡ Indicates *p* < 0.05 versus control group within the same level insulin sensitivity for the time trial and * indicates *p* < 0.05 between insulin-sensitive and insulin-resistant cells at the specified BCAA concentration. The gray dashed line in panel “a” represents the value of the insulin-sensitive control group at measurement 3 (18 min) as basal oxygen consumption for comparison across all groups. The green dashed line in panel “a” represents the value of the insulin-sensitive control group at measurement 9 (54 min) as FCCP-induced peak oxygen consumption for comparison across all groups. The red dashed line in panel “a” represents the value of the insulin-sensitive control group at measurement 12 (72 min) as rotenone-induced non-mitochondrial oxygen consumption for comparison across all groups. Images presented in panel “a” were captured using the 20× objective. Data in panels (**b**–**e**) were analyzed using two-way ANOVA with Bonferroni’s correction for multiple comparisons between individual groups. * Indicates *p* < 0.05 between insulin-sensitive and insulin-resistant cells at the specified BCAA concentration. Dissimilar letters indicate *p* < 0.05 between BCAA concentrations within the same level of insulin sensitivity.

Next, we assessed the effects of varied concentrations of BCAA both with and without insulin resistance on mitochondrial function. We observed significant reductions in both basal and FCCP-induced peak mitochondrial respiration in insulin-resistant cells versus their insulin-sensitive counterpart except for BCAA at 20 mM (Figure 2a). Interestingly, 20 mM BCAA depressed mitochondrial function in insulin-sensitive cells versus all other concentrations of BCAA (Figure 2a). These findings persisted for both basal and peak mitochondrial function after the removal of non-mitochondrial respiration and normalization to nuclei content (Figure 2b,c). After we observed reduced mitochondrial function in insulin-resistant cells and insulin-sensitive cells treated with 20 mM BCAA, we next assessed mitochondrial content via NAO staining. Like mitochondrial function, we observed significantly reduced mitochondrial content in insulin-sensitive cells treated with 20 mM (Figure 2d). This effect was exaggerated during insulin resistance, which resulted in reduced mitochondrial staining in cells treated with 0.2 mM, 2 mM, and 20 mM, suggesting BCAA coupled with insulin resistance leads to onset of reduced mitochondrial function and content at lower levels of BCAA. We also assessed the effect of each treatment on lipid content and observed that only insulin-sensitive cells treated with 20 mM BCAA exhibited lower lipid content (suggesting lipid availability was not likely responsible for reduced mitochondrial function) (Figure 2e).

**Figure 3:**
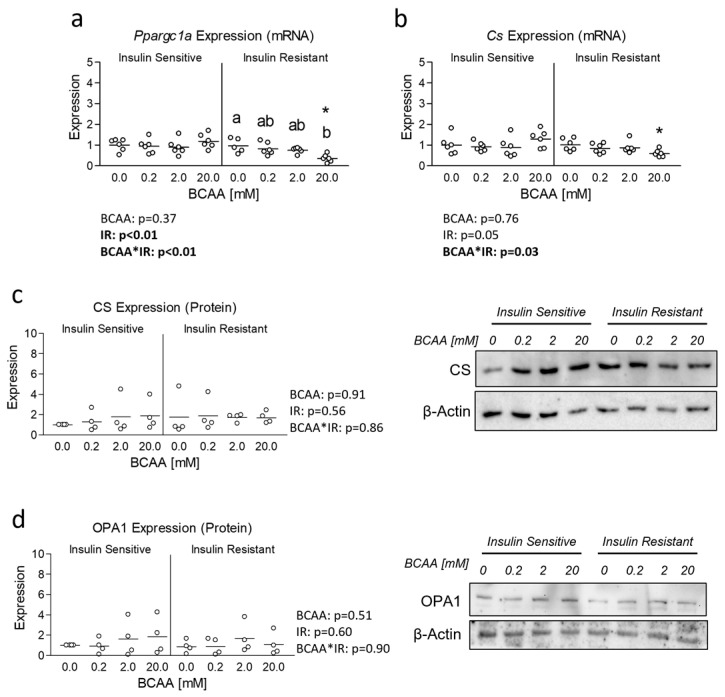
Effect of 6-day BCAA treatment with and without insulin resistance on mitochondrial biogenesis signaling. (**a**) Effect of BCAA 2:1:1 (leucine/isoleucine/valine) at varied concentrations (0, 0.2, 2, or 20 mM normalized to leucine content) for 6 days with and without insulin resistance (IR) on myotube mRNA expression of peroxisome proliferator-activated receptor-gamma coactivator-1alpha (Ppargc1a). (**b**) Effect of treatment as described in “a” on Citrate Synthase (Cs) mRNA expression. (**c**) Effect of treatment as described in “a” on Citrate Synthase (Cs) protein expression. (**d**) Effect of treatment as described in “a” on Optic atrophy 1 (OPA1) protein expression. Notes: Data were analyzed using two-way ANOVA with Bonferroni’s correction for multiple comparisons between individual groups. * Indicates *p* < 0.05 between insulin-sensitive and insulin-resistant cells at the specified BCAA concentration. Dissimilar letters indicate *p* < 0.05 between BCAA concentrations within the same level of insulin sensitivity. No statistical main effects were observed for BCAA treatment condition. Target gene expression was normalized to TATA binding protein (Tbp) using 3 replicates per group across 2 independent experiments with *n* = 5–6 for the final analysis. Protein expression was normalized to β-actin using 4 replicates per group with *n* = 4 for the final analysis.

To follow-up our examination of mitochondrial function, we next assessed the expression of the primary regulator of mitochondrial biogenesis, peroxisome proliferator-activated receptor gamma coactivator 1-alpha (Ppargc1a). We observed a significant main effect of insulin resistance in the suppression of Ppargc1a expression, which showed pairwise difference between insulin-resistant cells and insulin-sensitive cells at 20 mM BCAA (Figure 3a). We also observed a significant interaction effect between BCAA and insulin resistance, which revealed suppressed Ppargc1a expression in insulin-resistant cells treated with 20 mM BCAA versus insulin-resistant cells treated without additional BCAA (Figure 3a). We next assessed the expression of citrate synthase (Cs), a downstream target of Ppargc1a and indicator mitochondrial content. We again observed a significant interaction effect with a significant difference between insulin-resistant cells treated with BCAA at 20 mM and its insulin-sensitive BCAA-matched counterpart (Figure 3b). Though unlike reduced Ppargc1a expression in insulin-resistant cells treated with 20 mM BCAA, no differences between BCAA treated cells within each level of insulin sensitivity were observed (Figure 3b). Interestingly, neither treatment condition altered expression of CS at the protein level (Figure 3c). Similarly, no difference in the mitochondrial protein OPA1 was observed (Figure 3d).

### 3.3. Effect of BCAA Treatment on Mitochondrial Dynamics

**Figure 4:**
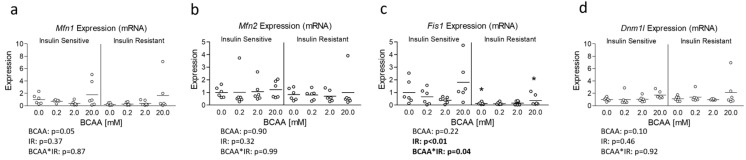
Effect of 6-day BCAA treatment with and without insulin resistance on mitochondrial dynamics-related mRNA expression. Effect of BCAA 2:1:1 (leucine/isoleucine/valine) at varied concentrations (0, 0.2, 2, or 20 mM normalized to leucine content) for 6 days with and without insulin resistance (IR) on myotube mRNA expression of (**a**) mitofusin 1 (Mfn1), (**b**) mitofusin 2 (Mfn2), (**c**) mitochondrial fission protein 1 (Fis1), and (**d**) Dynamin-1-like protein (Dnm1l). Notes: Data were analyzed using two-way ANOVA with Bonferroni’s correction for multiple comparisons. * Indicates *p* < 0.05 between insulin-sensitive and insulin-resistant cells at the specified BCAA concentration. No statistical main effects were observed for BCAA treatment condition. Target gene expression was normalized to TATA binding protein (Tbp) using 3 replicates per group across 2 independent experiments with *n* = 5–6 for the final analysis.

**Figure 5:**
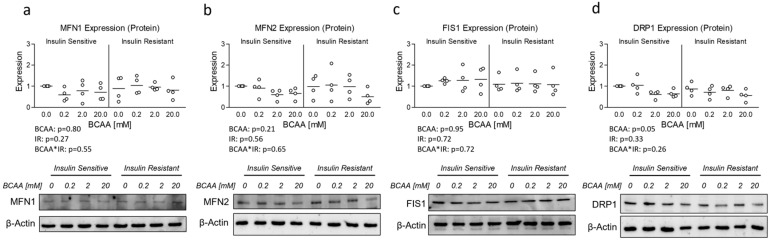
Effect of 6-day BCAA treatment with and without insulin resistance on mitochondrial dynamics-related protein expression. Effect of BCAA 2:1:1 (leucine/isoleucine/valine) at varied concentrations (0, 0.2, 2, or 20 mM normalized to leucine content) for 6 days with and without insulin resistance (IR) on myotube protein expression of (**a**) mitofusin 1 (MFN1), (**b**) mitofusin 2 (MFN2), (**c**) mitochondrial fission protein 1 (FIS1), and (**d**) Dynamin-related protein 1 (DRP1). Notes: Data were analyzed using two-way ANOVA. No statistical main effects were observed for BCAA treatment condition. Protein expression was normalized to β-actin using 4 replicates per group with *n* = 4 for the final analysis.

Lastly, although OPA1 protein expression was unaltered, insulin resistance has previously been associated with altered mitochondrial dynamics [10,11]. And because Ppargc1a is known to regulate several targets that govern mitochondrial dynamics [19] and was reduced in insulin-resistant cells treated with excess BCAA, we assessed the mRNA expression of several primary genes known to regulate mitochondrial dynamics. While no effect of insulin resistance or BCAA treatment was observed for Mitofusin 1 (Mfn1), Mitofusin 2 (Mfn2), or Dynamin-1-like protein (Dnm1l) also referred to as Dynamin-related protein 1 (Drp1) (Figure 4a, b, and d, respectively), a main effect for insulin resistance and significant interaction effect was observed for (Fis1), which was downregulated in insulin-resistant cells (Figure 4c). We next sought to assess if mRNA experiments paralleled protein expression of similar targets; however, we observed no main or interaction effects on protein expression of any mitochondrial dynamic targets (Figure 5).

## 4. Discussion

A perplexing relationship between BCAA and insulin resistance exists, with several proposed mechanisms previously discussed [1,2,3,4]. The possibility that excess BCAA reduces mitochondrial metabolism is one aspect of these potential mechanisms. It has also been postulated that accumulation of BCAA during insulin resistance results from down-regulation of BCAA-specific catabolic enzymes, which is also observed during diabetes [3,20,21]. We previously showed the coupling of insulin resistance with supraphysiological levels of BCAA had the most pronounced effect on mitochondrial function and related gene expression, though it should be noted that supraphysiological levels of BCAA (20 mM) also reduced mitochondrial function independent of insulin resistance [9]. We confirmed these findings within this report by showing that both basal and peak mitochondrial respiration were reduced in insulin-resistant cells or insulin-sensitive cells treated with supraphysiological levels of BCAA (20 mM). Importantly, like nuclei content/cell abundance during metabolism assays (Figure S1, to which metabolism/staining data were normalized), we also verified that reduced mitochondrial function was not a result of diminished cell viability (Figure S1b,c). And while subtle but significant differences between select groups were observed (Figure S1b,c), each BCAA-treated group appeared to have equal or higher viability than its respective control, suggesting BCAA-mediated cytotoxicity was not the cause of reduced mitochondrial function. Thus, observations of reduced mitochondrial function led us to further investigate the effects of BCAA and insulin resistance on mitochondrial dynamics, which could conceivably also contribute to altered mitochondrial function and reduced mitochondrial content. We initially hypothesized BCAA would increase fission-related signaling and decrease fusion-related signaling, which would be worsened by concurrent insulin resistance. However, we found that BCAA had no effects on any mitochondrial dynamic targets regardless of the presence of insulin resistance. And contrary to our original hypothesis, insulin resistance had no effect on the protein expression of mitochondrial fission proteins, though we did observe downregulated Fis1 at the mRNA level during insulin resistance. It is surprising that such reductions in mitochondrial function occurred without any change in mitochondrial dynamics. However, it could be that excess BCAA results in an accumulation of intermediates that impede mitochondrial function [22], and the changes in such metabolites may occur without substantive changes in mitochondrial dynamics (a speculation worthy of further investigation).

Interestingly, individual BCAA have been evaluated for their effects on mitochondrial content and function in various tissues [8]. Leucine is among the most heavily researched BCAA and has been associated with increased mitochondrial biogenesis, content, and/or function [5,8]. This has been shown across multiple in vitro experimental models of muscle including C2C12 myotubes [14,23,24,25,26,27,28,29], porcine skeletal muscle cells/muscle [30,31,32], and primary mouse myocytes [33]. Conversely, experiments with either isoleucine [33,34] or valine [28,33,34,35] have mostly shown a limited effect of either on mitochondrial function, though some evidence has shown valine may promote mitochondrial function and protect against reactive oxygen species [36]. In contrast to the above-mentioned in vitro reports, our experiments included a supraphysiological level of BCAA (20 mM), which was the concentration most associated with mitochondrial dysfunction. This concentration is far higher than many past experiments and may explain the disparities between the present report and other previous observations.

Collectively, our data indicate that the addition of BCAA did not alter indicators of mitochondrial dynamics regardless of the presence of insulin resistance. While insulin resistance modified expression of select mRNA targets associated with mitochondrial fission, insulin resistance did not alter the protein expression of any targets associated with altered mitochondrial dynamics. Taken together, our report provides among the first proof-of-concept experiments that have assessed the effect of BCAA on mitochondrial dynamics, both with and without insulin resistance. However, the in vitro model of our study makes it difficult to extrapolate to in vivo models. Additionally, we used cell culture media with sufficient BCAA content under basal (control) circumstances, which was selected because the complete void of BCAA is not physiologically relevant in the context of human disease. Another limitation of our experiment was the use of only one mediator of insulin resistance (hyperinsulinemic), which limits the comparability with other models of in vitro insulin resistance (such as palmitate). Similarly, we only assessed one indicator of insulin resistance (pAkt) following insulin stimulation; however, Akt is a target of insulin downstream of IRS1 and PI3K and is regarded as a central node in the proximal insulin signaling cascade [37]. Given that this model of insulin resistance has been shown to dose-dependently reduce Akt activation following insulin stimulation [24,38], and given that populations with insulin resistance often present with hyperinsulinemia [39,40], we felt it was an appropriate model of insulin resistance for this investigation. Thus, the implications of our study will require further investigation. These limitations aside, we believe our experiments provide a necessary first level of evidence on the effects of BCAA and insulin resistance on mitochondrial dynamics in skeletal muscle.

## 5. Conclusions

Elevated circulating BCAA consistently correlate with severity of insulin resistance, as does mitochondrial dysfunction. Our report demonstrates that excessively high BCAA reduces mitochondrial function as does insulin resistance in a myotube model of skeletal muscle. While indicators of mitochondrial biogenesis were reduced in insulin-resistant cells treated with supraphysiological levels of BCAA, BCAA did not alter indicators of mitochondrial dynamics regardless of the presence of insulin resistance. And while insulin resistance modified expression of select mRNA targets associated with mitochondrial fission, insulin resistance did not alter the protein expression of any targets associated with altered mitochondrial dynamics.

## Data Availability

The data that support the findings of this study are presented within the manuscript and supplemental material, and additional information is available from the corresponding author upon reasonable request.

## Supplementary Materials

The following supporting information can be downloaded at: https://www.mdpi.com/article/10.3390/metabo14070389/s1, Figure S1: DAPI staining and Cell viability; Table S1: qRT-PCR primers; Table S2: Western blot antibodies.
