# Supplementary material for: Excess Branched-Chain Amino Acids Suppress Mitochondrial Function and Biogenic Signaling but Not Mitochondrial Dynamics in a Myotube Model of Skeletal Muscle Insulin Resistance
Source: Metabolites. 2024 Jul 17;14(7):389. doi: 10.3390/metabo14070389 (PMC11279211; doi:10.3390/metabo14070389)
Supplement: Supplementary file 1 [file metabolites-14-00389-s001.zip › metabolites-3067930-supplementary.pdf]

Article (Supplemental Materials)

Excess branched-chain amino acids suppress mitochondrial function and biogenic signaling but not mitochondrial dynamics in a myotube model of skeletal muscle insulin resistance

Lindsey R. VanDerStad, Emily C. Wyatt, and Roger A. Vaughan *

Department of Health and Human Performance, High Point University, High Point, NC; lvanders@highpoint.edu, ewyatt@highpoint.edu, rvaughan@highpoint.edu

* Correspondence: rvaughan@highpoint.edu; Tel.: 1-336-841-9688

Supplemental Tables

Table s1 Summary of qRT-PCR primers.

Gene Abbreviation	Forward Sequence	Reverse Sequence
Cs	5'-TGAGAGGCATGAAGGGACTTGTGT-3'	5'-ATCTGTCCAGTTACCAGCAGCCAA-3'
Drp1	5'-TGCCTCAGATCGTCGTAGTG-3'	5'-TCTGGTGAAACGTGGACTAGC-3'
Fis1	5'-CAAAGAGGAACAGCGGGACT-3'	5'-CAACAGCCCTCGCACATACTT-3'
Mfn1	5'-CACTGCAATCTTCGGCCAGT-3'	5'-TTCTGGATTCCTGTATGTTGCTTCA-3'
Mfn2	5'-TGATGTGGCCCAACTCCAAG-3'	5'-GTAACATCGATCCCAGGGCTGT-3'
Ppargc1a	5'-GACAATCCCGAAGACACTACAG-3'	5'-AGAGAGGAGAGAGAGAGAGAGA-3'
Tbp	5'-GGGATTCAGGAAGACCACATA-3'	5'-CCTCACCAACTGTACCATCAG-3'

Abbreviations: Citrate Synthease (Cs), Dynamin-related protein 1 (*Drp1*), mitochondrial fission protein 1 (*Fis1*), mitofusin 1 (Mtfn1), mitofusin 2 (*Mfn2*), peroxisome proliferator-activated receptor-gamma coactivator-1alpha (*Ppargc1a*), Tata binding protein (*Tbp*).

Table s2 Summary of Western blot antibodies.

Protein Target	*Type*	*Dilution*	*Company*	*Item*	*Approx. Mol Wt.*	*Product Link*
pAkt (Ser 473)	RP	1:1000	SC Biotechnology	sc-7985-R	62kd	p-Akt1/2/3 (Ser 473)
Akt	RP	1:1000	Cell Signaling	9272	62kd	Akt Antibody#9272
β-Actin	MM	1:500	SC Biotechnology	sc-47778	43kd	Datasheet
CS	MM	1:1000	SC Biotechnology	sc-390693	52kd	sc-390693
DRP1	RP	1:1000	ProSci	7603	80kd	DNM1L Antibody
FIS1	RP	1:1000	Novus Biologicals	NB100-56646	17kd	TTC11 Antibody (NB100-56646)
MFN1	RP	1:1000	ProSci	7861	83kd	MFN1 Antibody
MFN2	RP	1:1000	ProSci	7863	83-90kd	MFN2 Antibody
OPA1	RM	1:1000	Cell Signaling	80471	88-100kd	OPA1 (D6U6N) Rabbit mAb

Abbreviations: Citrate synthase (CS), dynamin-related protein 1 (DRP1), mitochondrial fission protein 1 (FIS1), mitofusin 1 (MFN1), mitofusin 2 (MFN2), mouse monoclonal (MM), optic atrophy 1 (OPA1), rabbit monoclonal (RM), and rabbit polyclonal (RP). Notes: Target molecular weight was based on product datasheet. Molecular weights for all targets were verified against sizes suggested by product brochures.

Supplemental Figure

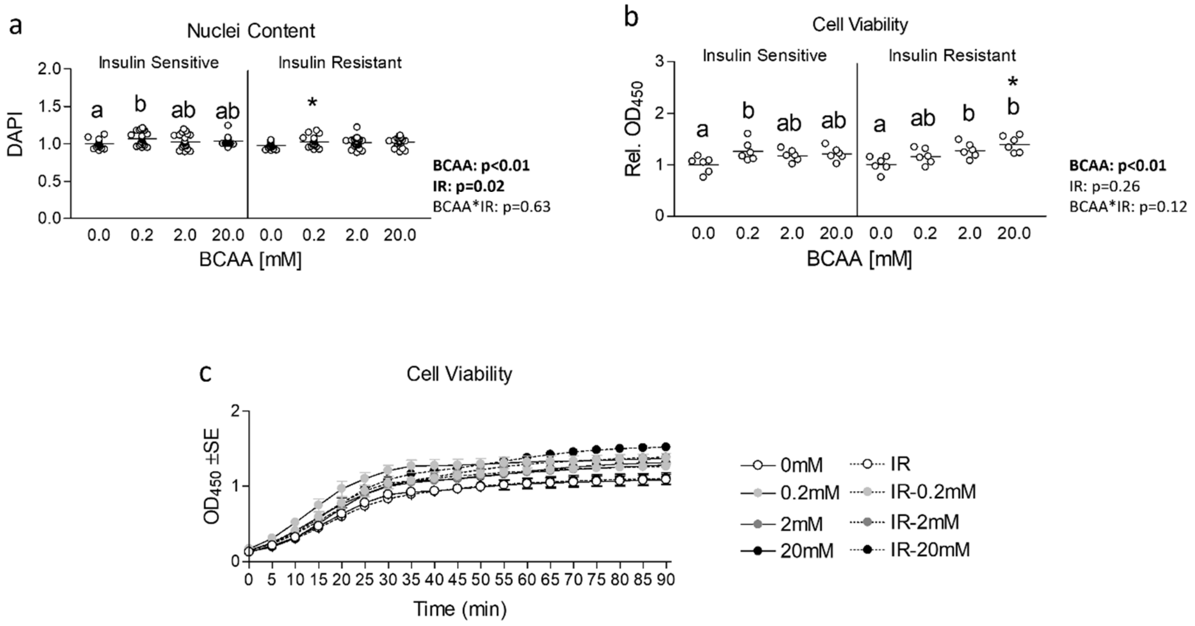

Figure s1 Effect of 6-day BCAA on nuclei content and viability. (a) Nuclei content from Seahorse Assay following treatment of myotubes with BCAA 2:1:1 (leucine:isoleucine:valine) at varied concentrations (0, 0.2, 2, or 20mM normalized to leucine content) for 6 days with and without insulin resistance (IR). (b and c) Cell viability following treatment as described in "a" expressed as (b) relative viability end point and (c) complete viability trial.

Notes: Data were analyzed using two-way ANOVA with Bonferroni's correction for multiple comparisons. * Indicates $p<0.05$ between insulin sensitive and insulin resistant cells at the specified BCAA concentration. Dissimilar letters indicate $p<0.05$ between BCAA concentrations within the same level of insulin sensitivity. DAPI staining was from n=11-12 per group repeated with 2 independent experiments for n=22-24 per group for the final analysis. Cell viability was from n=3 per group repeated with 2 independent experiments for n=6 per group for the final analysis.
